# Erk1/2 Inactivation-Induced c-Jun Degradation Is Regulated by Protein Phosphatases, UBE2d3, and the C-Terminus of c-Jun

**DOI:** 10.3390/ijms22083889

**Published:** 2021-04-09

**Authors:** Weiming Ouyang, David M. Frucht

**Affiliations:** Division of Biotechnology Review and Research II, Office of Biotechnology Products, Office of Pharmaceutical Quality, Center for Drug Evaluation and Research, U.S. Food and Drug Administration, Silver Spring, MD 20993, USA

**Keywords:** ubiquitin E3 ligase, COP1, protein phosphatase, UBE2d3, c-Jun, protein degradation

## Abstract

Constitutive photomorphogenic 1 (COP1) is the ubiquitin E3 ligase that mediates degradation of c-Jun protein upon Erk1/2 inactivation. It remains unknown how this protein degradation pathway is regulated. In this study, we investigated the roles of protein phosphatases, ubiquitin-conjugating E2 enzymes (UBE2), and an intrinsic motif of c-Jun in regulating this degradation pathway. By using pharmacological inhibitors and/or gene knockdown techniques, we identified protein phosphatase 1 (PP1) and PP2A as the phosphatases and UBE23d as the UBE2 promoting c-Jun degradation, triggered by Erk1/2 inactivation. In addition, we report that the C-terminus of c-Jun protein facilitates its degradation. The addition of a C-terminal tag or deletion of the last four amino acid residues from the C-terminus of c-Jun protects it from degradation under Erk1/2-inactivating conditions. Taken together, this study reveals that the Erk1/2 inactivation-triggered and COP1-mediated c-Jun degradation is extrinsically and intrinsically regulated, providing a new understanding of the mechanisms underlying this protein degradation pathway.

## 1. Introduction

c-Jun is a key member of the activation protein 1 (AP-1) transcription factor family, which regulates numerous cellular activities [1,2]. c-Jun is degraded by the ubiquitination-dependent proteasome pathway [3]. Studies from our laboratory have revealed that inactivation of the MKK1/2–Erk1/2 signaling pathway by chemical MKK1/2 inhibitors or anthrax lethal toxin (LT) reduces levels of the c-Jun protein rapidly by promoting its degradation via a COP1-dependent pathway [4,5].

COP1 was originally identified during the study of the *COP/DET/FUS* loci in plants, in which it was found to function as a ubiquitin E3 ligase that promotes the degradation of photoreceptors and photomorphogenic transcription factors such as HY5, HYH, LAF1, and HFR1, thereby repressing plant photomorphogenesis. [6,7]. In mammalian cells, COP1 serves as a ubiquitin E3 ligase for c-Jun [8,9], ETS transcription variants (ETVs) [10,11,12], p53 [13], and COP1 itself [14]. At a steady state, COP1 is sequestered on the nuclear envelope, allowing the accumulation of transcription factors that are required for cellular proliferation. MKK1/2–Erk1/2 inactivation leads to a rapid release of COP1 from sequestration [5], thereby promoting the degradation of COP1-targeted transcription factors in the nucleus, leading to cell growth arrest [5]. Our previous study suggested that the sequestration of COP1 requires Erk1/2-mediated phosphorylation of its binding partners, such as translocated promoter region (TPR) [5]. The phosphorylation status of these COP1-binding partners is co-regulated by protein kinases and protein phosphatases. However, it remained unknown whether one or more protein phosphatase(s) have a role in regulating Erk1/2 inactivation-triggered and COP1-dependent protein degradation pathway.

As an E3 ligase, COP1 plays a major role in the protein ubiquitination process, which is a cascade catalyzed by three major enzyme components—a ubiquitin-activating enzyme (E1), a ubiquitin-conjugating enzyme (E2), and a ubiquitin ligase (E3) [15,16]. The process of protein ubiquitination starts with activation of the ubiquitin (Ub) C-terminus by an E1 in an ATP-dependent reaction to generate a thioester-linked E1~Ub conjugate. The activated Ub is then transferred to an E2 enzyme via a trans-thiolation reaction. Finally, an E3 ligase binds both a substrate and the E2~Ub conjugate, mediating the transfer of Ub most commonly onto the ε-amino group of a lysine in the target protein. There are approximately 35 E2s in mammals [17]. Although Ubc5Hb (Ube2d2) has been shown to act as an E2 for COP1-mediated ubiquitination in cell-free systems in vitro [9,14,18], it was not known whether this E2 also mediates Erk1/2 inactivation-induced c-Jun degradation in cells.

In this study, we addressed these unanswered questions regarding COP1-dependent c-Jun degradation, having identified PP1 and PP2A as the phosphatases and Ube2d3 as the E2 that promote c-Jun degradation by this pathway. In addition, we report that the C-terminus of c-Jun protein plays an intrinsic role in regulating its degradation following Erk1/2 inactivation.

## 2. Results

### 2.1. Protein Phosphatases Promote c-Jun Protein Degradation in Erk1/2 Inactivating Conditions

**Figure 1:**
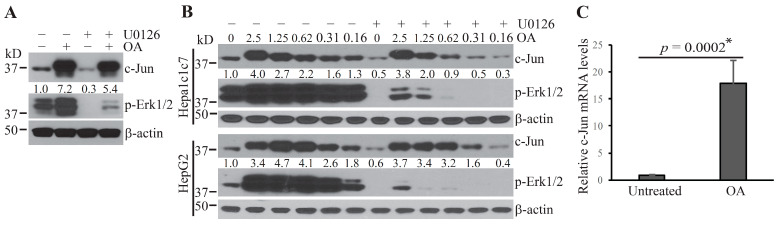
Protein phosphatase inhibition increases the levels of c-Jun protein. (**A**) Hepa1c1c7 cells were pretreated with or without 2.5 µM okadaic acid (OA) for 30 min and then incubated with or without 10 µM U0126 for 1 h. (**B**) Hepa1c1c7 and HepG2 Cells were pretreated with serially diluted concentrations of OA as indicated for 30 min and then treated with or without U0126 for 1 h. Cells were extracted with NuPAGE lithium dodecyl sulfate (LDS) sample buffer following the treatment. Protein levels of c-Jun, p-Erk1/2, and β-actin in the cell lysates were assessed by Western blotting. Band intensities of c-Jun were quantified and normalized using β-actin levels. The normalized amounts are shown under each band. Data shown are representative of at least two independent experiments. (**C**) Hepa1c1c7 cells were treated with 2.5 µM OA for 1.5 h. c-Jun mRNA levels were measured by qPCR and normalized using levels of *Gapdh*. Data shown were generated from three sets of independently prepared samples assessed by qPCR twice. Data were statistically analyzed using an unpaired, two-tailed Student’s *t*-test, and significant difference * was determined to be *p* < 0.01.

Our previous study showed that the ubiquitin E3 ligase, COP1, is sequestered and maintained in a poised status under steady-state conditions [5]. Erk1/2 inactivation following treatment with anthrax LT or the MKK1/2 inhibitor, U0126, induces rapid activation of COP1, leading to the degradation of its substrates, c-Jun, and ETV proteins [5]. The sequestration of COP1 is dependent on the Erk1/2-mediated phosphorylation of COP1-binding partners. Since the phosphorylation states are generally determined by the net balance between the activities of kinases and phosphatases [19], we hypothesized that phosphatase(s) may play a critical role in regulating the process leading to COP1 activation and degradation of its substrates upon MKK1/2–Erk1/2 inactivation. To test this hypothesis, we pretreated Hepa1c1c7 cells with the protein phosphatase inhibitor, okadaic acid (OA), and then cultured the cells with U0126 to inactivate the MKK1/2–Erk1/2 signaling pathway. Pretreatment of the cells with OA dramatically increased the baseline level of c-Jun protein and prevented c-Jun degradation following U0126 treatment (Figure 1A). To characterize further the ability of OA to prevent c-Jun degradation, we pretreated Hepa1c1c7 and HepG2 cells with serially diluted concentrations of OA and then cultured the cells with U0126. As shown in Figure 1B, pretreatment with OA increased the levels of c-Jun protein and prevented its degradation following U0126 treatment in both cell types in a dose-dependent manner.

OA has been reported to increase c-Jun gene transcription in U937 cells following OA treatment for 3 h. To investigate the potential effect of OA on c-Jun gene transcription in Hepa1c1c7 cells, we treated the cells with OA for 1.5 h and measured the levels of *c-Jun* mRNA by quantitative PCR. Unexpectedly, the *c-Jun* mRNA level was markedly increased following 1.5 h OA treatment (Figure 1C). These results suggest that the increase in c-Jun protein in OA-treated cells may due to both enhancement of c-Jun gene transcription and blockade of c-Jun protein degradation.

**Figure 2:**
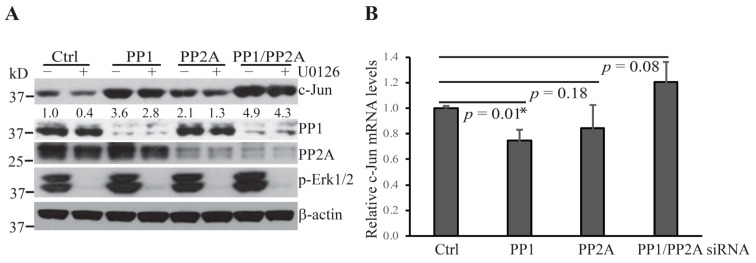
Protein phosphatases PP1 and PP2A promote c-Jun degradation following U0126 treatment. (**A**) Hepa1c1c7 cells were transfected with the indicated siRNA targeting PP1 and/or PP2A. Two days following transfection, the cells were cultured with or without U0126 for 1 h and then extracted with NuPAGE LDS sample buffer. The expression levels of the indicated proteins in the cell lysates were assessed by Western blotting. Knockdowns of target proteins by siRNAs were confirmed at the protein level by Western blotting. Band intensities of c-Jun were quantified and normalized using β-actin levels. The normalized protein levels are shown under each band. Data shown are representative of three independent experiments. (**B**) Two days following transfection of PP1 and/or PP2A siRNA, Hepa1c1c7 cells were extracted with TRIzol for RNA preparation. *c-Jun* mRNA levels were measured by qPCR and normalized by the levels of *Gapdh*. Data shown were generated from two sets of independently prepared samples assessed by qPCR twice. Data were statistically analyzed using an unpaired, two-tailed Student’s *t*-test and significant difference * was determined by *p* < 0.01.

OA inhibits the activity of the Ser/Thr phosphatases, PP1 and PP2A, which have been shown to localize to the nuclear envelope and the nucleus [20,21,22]. These phosphatases were of special interest since COP1 localizes and functions to promote the degradation of its substrates in the nucleus [5,14]. To investigate whether these protein phosphatases are involved in the regulation of Erk1/2 inactivation-induced c-Jun degradation, we used a standard siRNA technique to knock down the expression of PP1 and PP2A. Hepa1c1c7 cells were transfected with siRNA specifically targeting the PP1 and/or PP2A gene expression. In addition, 48 h following the initiation of the transfection, cells were treated with U0126. Knockdown of either PP1 or PP2A increased the levels of c-Jun protein and rescued it from U0126-induced degradation (Figure 2A). In contrast to OA treatment, knockdown of PP1 or PP2A individually did not increase the levels of c-Jun mRNA (Figure 2B), suggesting that these protein phosphatases act to reduce c-Jun protein levels upon Erk1/2 inactivation mainly by promoting c-Jun degradation. The increase in c-Jun levels and the rescue of c-Jun from U0126-induced degradation was more profound following PP1 knockdown than PP2A knockdown, and combined PP1/PP2 knockdown resulted in an additive rescuing effect. Moreover, PP1 and/or PP2 knockdown did not reverse U0126-induced Erk1/2 dephosphorylation, indicating that PP1 and PP2A act on other regulators of the COP1 activation cascade instead.

### 2.2. Ube2d3 Is the E2 Essential for Erk1/2 Inactivation-Induced c-Jun Protein Degradation

**Figure 3:**
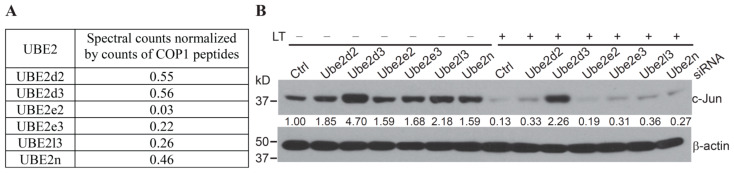
Ube2d2 functions as the E2 for COP1-mediated c-Jun degradation following anthrax lethal toxin (LT) treatment. (**A**) UBE2 in COP1 immunoprecipitated complex. Data shown are the relative spectral counts of UBE2s normalized by the spectral counts of COP1 peptides. (**B**) Hepa1c1c7 cells were transfected with the siRNA targeting the indicated Ube2s. Two days following transfection, the cells were cultured with or without LT for 2 h and then extracted with NuPAGE LDS sample buffer. Protein levels of c-Jun and β-actin in the cell lysates were assessed by Western blotting. Band intensities of c-Jun were quantified and normalized using β-actin levels. The normalized protein levels are shown under each band. Data shown are representative of three independent experiments.

COP1 is the ubiquitin E3 ligase that mediates c-Jun degradation under Erk1/2-inactivating conditions. However, in addition to an E3, the process of protein ubiquitination requires an E1 and an E2 [15,16]. Mass spectrometry analysis of the COP1 immunoprecipitated complex revealed that Ube2d2, Ube2d3, Ube2e2, Ube2e3, Ube2l3 and Ube2n co-precipitated with COP1 (Figure 3A). Of these, Ubc5Hb (Ube2d2) has been demonstrated to be an E2 for COP1-mediated ubiquitination of c-Jun and p53 in cell-free systems in vitro [9,14,18]. However, it had not been determined whether Ube2d2 also functions as a specific E2 for Erk1/2 inactivation-induced COP1-mediated c-Jun degradation in living cells. To identify the E2 involved in the process of Erk1/2 inactivation-induced and COP1-dependent c-Jun degradation, we knocked down the expression of a panel of COP1-associated E2s in Hepa1c1c7 using siRNA. We then examined c-Jun degradation following treatment with anthrax LT, which blocks the MKK1/2–Erk1/2 signaling by cleavage of MKK1 and MKK2 [4,23,24]. Knockdown of Ube2d3 increased the level of c-Jun protein at a steady state and markedly reduced c-Jun degradation in LT-treated cells. Although knockdowns of Ube2d2 and other tested E2s slightly increased the levels of c-Jun in cells in steady-state conditions, they failed to prevent c-Jun degradation following LT treatment (Figure 3). These results indicate that Ube2d3 functions as the specific E2 that promotes c-Jun degradation in cells following MKK1/2–Erk1/2 inactivation.

### 2.3. The C-Terminus of c-Jun Protein Regulates Its Degradation under Erk1/2 Inactivating Conditions

**Figure 4:**
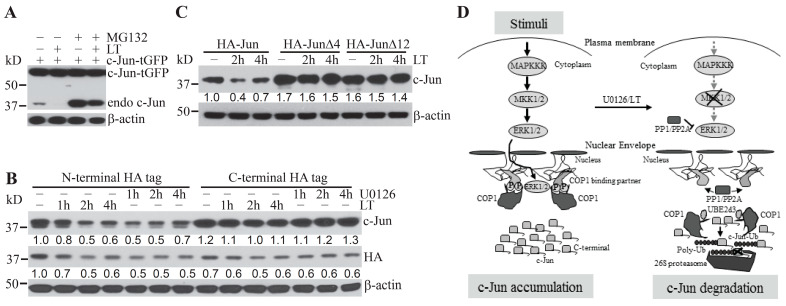
Regulation of c-Jun degradation by its distal C-terminus and a schematic model for the regulation of COP1-mediated c-Jun degradation. (**A**) Hepa1c1c7 cells transiently expressing c-Jun-tGFP were pretreated with 10 µM MG132 for 30 min and then cultured with or without LT for 2 h. (**B**,**C**) Hepa1c1c7 cells were transduced with pseudo retroviruses expressing c-Jun with either an HA-tag at its N-terminus or at its C-terminus (**B**), N-terminal HA-tagged full-length c-Jun, or c-Jun with a deletion of its C-terminal 4 (HA–JunΔ4) or 12 (HA–JunΔ12) amino acids (**C**). Hepa1c1c7 cells transduced with the indicated c-Jun constructs were treated with U0126 or LT as indicated. Cells (**A**–**C**) were extracted with NuPAGE LDS sample buffer following the treatment. Levels of the indicated proteins in the cell lysates were assessed by Western blotting. Band intensities of c-Jun were quantified and normalized using β-actin levels (**B**,**C**). The normalized protein levels are shown under each band. Data shown are representative of at least two independent experiments. (**D**) Extracellular stimuli activate the MAPKKK–MKK1/2–Erk1/2 signaling cascade. Activated Erk1/2 then induces the phosphorylation of COP1-binding partners, promoting sequestration of COP1 and accumulation of c-Jun protein (left panel). Deprivation of extracellular stimuli or inhibition of MKK1/2 by U0126/LT causes deactivation of Erk1/2 and dephosphorylation of COP1-binding partners, leading to the release of COP1. COP1, in turn, mediates c-Jun degradation by proteasome through a mechanism dependent upon UBE2d3- and the C- terminus of c-Jun (right panel).

Human and murine c-Jun proteins contain 331 and 334 amino acid residues, respectively. Both proteins include the EEPQTVPE motif, which is a consensus COP1-binding motif [D/E](x)xxVP[D/E] that mediates the binding of COP1 substrates to the WD40 domain of COP1 protein [10]. It was not previously clear whether other regions besides the COP1-binding motif in c-Jun also regulate Erk1/2 inactivation-induced and COP1-mediated c-Jun protein degradation. We unexpectedly observed that, in contrast to experiments involving endogenous c-Jun, levels of ectopically over-expressed c-Jun incorporating a C-terminal tGFP tag were not modulated by treatment with the proteasome inhibitor, MG132, and/or the Erk1/2 activation inhibitor, LT (Figure 4A). These results indicate that c-Jun-tGFP is resistant to proteasome-mediated protein degradation via the Erk1/2 inactivation-induced and COP1-dependent degradation pathway.

We next explored whether the resistance of c-Jun-tGFP could be caused by a confounding effect resulting from the alteration of its C-terminal conformation following the fusion to the relatively large-sized tGFP tag. To this end, we generated stable Hepa1c17 cell lines expressing c-Jun fusion proteins with either a small HA-tag at the N-terminus or the C-terminus. Following treatment with LT or U0126, the levels of c-Jun with the N-terminal tag exhibited markedly greater reductions than the levels of c-Jun with a C-terminal HA tag (Figure 4B). Consistently, the total c-Jun levels in Hepa1c1c7 cells stably expressing c-Jun with the N-terminal HA tag were markedly reduced in a time-dependent manner (Figure 4B). In contrast, total c-Jun levels in Hepa1c1c7 cells stably expressing c-Jun with the C-terminal HA tag showed very little change following the treatment (Figure 4B).

As the HA tag contains only nine amino acid residues, it is unlikely to cause a drastic change in the conformation of c-Jun C-terminus, suggesting that the C-terminus may have an intrinsic role in regulating c-Jun stability. We next generated stable cell lines expressing N-terminally HA-tagged full-length c-Jun (HA–Jun) or c-Jun with deletions of its last four residues (HA–JunΔ4) or its last 12 residues (HA–JunΔ12). Following treatment with LT, the c-Jun protein levels in cells ectopically expressing the HA–Jun control, but not HA–JunΔ4 and HA–JunΔ12, were markedly reduced (Figure 4C). These results confirmed that a discrete region of the C-terminus of c-Jun protein plays an intrinsic role in regulating its degradation upon MKK1/2–Erk1/2 inactivation.

## 3. Discussion

c-Jun protein is rapidly degraded by a COP1-dependent pathway in cells upon MKK1/2–Erk1/2 inactivation [5]. In this study, we report that the phosphatases, PP1 and PP2A, and the E2 enzyme, Ube2d3, promote c-Jun degradation upon inhibition of the MKK1/2–Erk1/2 signaling pathway (Figure 4D). In addition, we have identified a discrete region in the C-terminus of c-Jun protein that plays a critical role in regulating its degradation under Erk1/2 inactivating conditions (Figure 4D).

COP1 is sequestered and maintained in a functionally poised status under steady-state conditions, which is dependent upon Erk1/2 activity [5]. Erk1/2 activity, in turn, requires MKK1/2, which catalyzes the phosphorylation of tyrosine and threonine residues in the Erk1/2 proteins and active their enzymatic activities [25]. Activated Erk1/2 catalyzes the phosphorylation of hundreds of cytoplasmic and nuclear substrates, including regulatory molecules and transcription factors that contain the Pro-Xxx-Ser/Thr-Pro motif [25]. When MKK1/2 is inhibited by an inhibitor or cleaved by LT, Erk1/2 and its substrates are rapidly dephosphorylated by protein phosphatases. PP1 and PP2A are protein phosphoserine/phosphothreonine-specific protein phosphatases belonging to the phosphoprotein phosphatases (PPPs) family. They localize to the nucleus and nuclear periphery [22,26], in the same region where COP1 is poised to promote the degradation of its substrates [5,14]. Our data are consistent with a model in which protein phosphatases rapidly dephosphorylate Erk1/2 and COP1-binding partners, leading to activation of COP1 and degradation of its substrates, including c-Jun.

The phosphatase inhibitor, OA, which targets PP1 and PP2A, is effective in blocking COP1-dependent c-Jun degradation. OA has previously been reported to activate c-Jun gene transcription, leading to increased c-Jun mRNA levels in U937 cells, but this effect was noted no earlier than 3 h following the treatment with OA [27]. Unexpectedly, in our experimental system, *c-Jun* mRNA levels were significantly increased in Hepa1c1c7 cells following 1.5-h OA treatment, suggesting that the rapid increase in c-Jun protein levels following OA treatment may be due to both increased *c-Jun* transcription and blockade of COP1-dependent c-Jun degradation. In contrast to OA treatment, knockdown of the expression of PP1 or PP2A individually did not increase the levels of *c-Jun* mRNA, indicating that PP1 and PP2A may act to reduce the levels of c-Jun protein upon Erk1/2 inactivation mainly by promoting c-Jun degradation. Interestingly, knockdown of both PP1 and PP2A slightly increased the levels of c-Jun, which is in line with the finding observed in OA-treated cells. These findings suggest that protein phosphatases regulate c-Jun at both gene transcription and protein stability levels. Understanding the mechanism by which PP1 and PP2A regulate *c-Jun* transcription warrants further investigation.

Enhancement of baseline Erk1/2 phosphorylation and maintenance of a portion of Erk1/2 phosphorylation following blockade of MKK1/2-dependent signal transduction by U0126 were observed in OA-treated Hepa1c1c7 cells, but not in Hepa1c1c7 cells following PP1 and/or PP2A knockdown. These findings suggest that PP1 and PP2A act on other regulators of the Erk1/2 and COP1-dependent c-Jun degradation pathway instead. Identification of these specific regulators warrants further investigation. Interestingly, PP1 knockdown showed a more profound effect than PP2A knockdown on the increase in c-Jun levels and the rescue of c-Jun from U0126-induced degradation. However, PP2A is very abundant in the cells, and its knockdown is less efficient as compared to PP1. Therefore, future experiments using PP1 and PP2 knockout cell lines will be required to confirm whether PP1 acts as the dominant protein phosphatase in the regulation of the process of c-Jun degradation triggered by inhibition of the MKK1/2–Erk1/2 signaling pathway.

In addition to the identification of upstream regulators of COP1-dependent c-Jun degradation, we have also elucidated an essential downstream component of this pathway. As previously reported, this process depends on the ubiquitin E3 ligase, COP1, and proteasome activity [4,5]. In contrast to previous results generated from in vitro cell-free systems, we identify Ube2d3, but not Ube2d2/Ubc5Hb, as an E2 that mediates COP1-mediated ubiquitination [14,18] and promotes c-Jun degradation upon inactivation of the MKK1/2–Erk1/2 signaling pathway in living cells. Nevertheless, it is important to note that in addition to COP1, other ubiquitin E3 ligases including FBW7 [28], ITCH [29], and MEKK1 [30] can also function as an E3 ligase to promote c-Jun degradation. Our experiments demonstrated that knockdown of Ube2d2, Ube2e2, Ube2e3, Ube2l3, and Ube2n slightly increase the levels of c-Jun protein at a steady state, suggesting these E2s may work with other E3 ligases to regulate c-Jun stability via different pathways.

Ultimately, COP1-dependent c-Jun degradation following ERK1/2 inactivation is executed by the 26S proteasome, which can be blocked by pretreatment with the proteasome inhibitor, MG123 [4]. This process is dependent upon a consensus COP1-binding motif [D/E](x)xxVP[D/E] on c-Jun, which binds the WD40 domain of COP1 protein. In this study, we identified another critical region in the C-terminus of c-Jun protein that plays an intrinsic role in regulating its degradation. We hypothesize that this region is involved in the targeting of c-Jun to the 26S proteasome. The 26S proteasome contains two major assemblies—the 28-subunit core particle (CP, also known as the 20S particle) and a regulatory particle of 19–20 subunits (RP, also known as the 19S particle) [31,32,33]. The CP is a barrel-like structure, with its subunits arranged in four stacked seven-membered rings with proteolytically active sites within the large internal space. The RP binds to the cylinder end of the CP, opens a channel located centrally within the cylinder, and guides substrates into this channel during protein degradation. During this process, the RP plays an essential role in the recognition, unfolding, translocation, and de-ubiquitination of the substrate. The attached K48-polyubiquitin chain marks a protein for degradation. However, an unstructured region near the end of the substrate polypeptide is also required for efficient proteasome degradation of the substrate [34,35,36]. The attached poly-ubiquitin chain undergoes reversible binding to ubiquitin receptors associated with the RP, followed by an unstructured region-dependent binding to the RP, which leads to unfolding and destruction of the substrate. The C-terminus of c-Jun that we have identified likely functions as the unstructured region facilitating tight binding to the RP, thereby promoting proteasomal c-Jun degradation.

As a key member of the AP-1 transcription factor family, c-Jun has a critical role in regulating numerous cellular activities, including cellular proliferation, differentiation, survival, death, tumorigenesis, and T cell exhaustion [1,2,37,38,39]. We have also previously reported that COP1-mediated c-Jun degradation may play a pathogenic role during anthrax infection, which causes cell cycle arrest and inhibition of the proliferation of vascular endothelial cells, intestinal progenitor cells, T cells, and B cells [40,41,42,43], leading to vascular vessel leakage and breakdown of the intestinal barrier and adaptive immune system. Identification of the components of the COP1-mediated c-Jun degradation pathway provides additional mechanistic insights and potential therapeutic targets for a variety of clinical diseases in which c-Jun plays a role in pathogenic mechanisms.

## 4. Materials and Methods

### 4.1. Cells and Reagents

Murine liver hepatoma Hepa1c1c7 (CRL-2026™), human hepatocellular carcinoma HepG2 (HB-8065), and human embryonic kidney 293T (CRL-11268™) cells used in this study were purchased from the American Type Culture Collection (ATCC, Manassas, VA). Hepa1c1c7 cells were cultured in an alpha minimum essential medium, HepG2 cells were cultured in Eagle’s minimum essential medium, and 293T cells were cultured in Dulbecco’s modified Eagle’s medium. All media were supplemented with 10% FBS, 100 IU/mL penicillin, 100 µg/mL streptomycin, and 2 mM L-glutamine. Cultures were maintained at 37 °C in an incubator with 5% CO_2_. Lyophilized recombinant protective antigen (PA) and lethal factor (LF) were purchased from List Biological Laboratories, Inc., and reconstituted in sterile water to make stock solutions with final concentrations of 1 mg/mL. Okadaic acid (OA), calyculin A (CA), MG132 (inhibitor of 26 S proteasome), and U0126 (inhibitor of MKK1/2) were purchased from EMD Millipore. Unless specified, OA, CA, U0126, and MG132 were used to treat cells at concentrations of 2.5 µM, 100 nM, 10 µM, and 10 µM, respectively. For the LT treatment, cells were cultured with 2 µg/mL PA and 1 µg/mL LF. NuPAGE LDS sample buffer (4×) was obtained from ThermoFisher Scientific.

### 4.2. Plasmids and Cell Transfection

The pCMV6-AC-GFP-c-Jun plasmid was purchased from Origene. This construct expresses c-Jun with the turboGFP (tGFP) tag at its C-terminal. cDNA sequences encoding the full-length c-Jun or c-Jun with deletion of the last 4 and 12 C-terminal amino acid residues were amplified by PCR using the high fidelity PrimeSTAR Max DNA Polymerase (Takara, Mountain View, CA) and cloned into the TA vector pDRIVE (Qiagen, Germantown, MD). The c-Jun-encoding fragments were released and inserted into the retroviral pMIG-w vector containing an N-terminal or C-terminal HA tag. All DNA sequences were confirmed by DNA sequencing after cloning. To produce pseudo-retrovirus, retroviral plasmids were transfected into 293T cells, together with packaging plasmids, using a standard calcium phosphate method. Supernatants were harvested 48 h following the transfection, filtered through 0.45 µm filters (Millipore), and then used to transduce cells. siRNA was transfected into cells using Lipofectamine RNAiMAX reagent (Invitrogen, Waltham, MA), following the manufacturer’s suggested protocol. siRNAs were all purchased from ThermoFisher Scientific, and the ID number of the siRNAs used in this study are listed as follows: negative control (4390843), PP1 (HSS143413), PP2A (S10958), Ube2d2 (S14575), Ube2d3 (S82582), Ube2e2 (S104367), Ube2e3 (S75661), Ube2l3 (S75668) and Ube2n (S123786).

### 4.3. Mass Spectrometry

Approximately 200 million Hepa1c1c7-HA-COP1 cells harvested from 10 15-cm plates were used to prepare the nucleus extracts using the Nuclear Complex Co-IP Kit from Active Motif. The nuclear extracts were incubated with anti-HA magnetic beads following the manufacturer’s instructions (ThermoFisher Scientific cat#88837) to precipitate the COP1 complex. The immunoprecipitated complex was digested on beads and then analyzed by mass spectrometry using the Orbitrap Fusion instrument at the Mass Spectrometry and Proteomics Facility of Ohio State University. Sequence information from the MS/MS data was processed by converting the raw files into a merged file using MSConvert (ProteoWizard). Isotope distributions for the precursor ions of the MS/MS spectra were deconvoluted to obtain the charge states and monoisotopic m/z values of the precursor ions during the data conversion. The resulting mgf files were searched using Mascot Daemon by Matrix Science version 2.5.1 (Boston, MA, USA), and the database was searched against the most recent Uniprot databases. A decoy database was also searched to determine the false discovery rate (FDR), and peptides were filtered according to 1% FDR. Proteins identified with at least two unique peptides were considered as reliable identification. Any modified peptides were manually checked for validation.

### 4.4. Quantitative PCR

Total RNA was extracted from cells with TRIzol (Invitrogen) following the manufacturer’s suggested protocol. RNA was then reverse transcribed to cDNA using the Omniscript RT kit manufactured by Qiagen (Valencia, CA). The levels of c-Jun mRNA were measured by quantitative PCR performed using standard techniques with a TaqMan probe for murine *c-Jun* (Mm00495062_s1) purchased from Invitrogen and normalized to the levels of *Gapdh* (Mm99999915_g1). Statistical analyses were performed using the unpaired, two-tailed Student’s *t*-test.

### 4.5. Western Blotting

Cells were lysed with NuPAGE LDS sample buffer (Invitrogen). Cell lysates were then separated on 4–12% NuPAGE BisTris gels (Invitrogen) and transferred to nitrocellulose membranes (Bio-Rad, Hercules, CA, USA). The membranes were probed with antibodies of interest using standard Western blotting techniques as described previously [4,5,24]. Antibodies targeting c-Jun, p-Erk1/2, HA tag, PP1α, PP2A, and β-actin were purchased from Cell Signaling (Danvers, MA, USA). The intensities of protein bands were quantified using the Image Studio software, supplied with the Odyssey system, and normalized to the amounts of β-actin that were used as loading controls.

## Data Availability

Data available on request.
